# Interspecific variation of antennal lobe composition among four hornet species

**DOI:** 10.1038/s41598-021-00280-z

**Published:** 2021-10-22

**Authors:** Antoine Couto, Gérard Arnold, Hiroyuki Ai, Jean-Christophe Sandoz

**Affiliations:** 1 Laboratory Evolution Genomes Behavior and Ecology, CNRS, University Paris-Sud, IRD, Université Paris Saclay, 1 avenue de la Terrasse, 91198 Gif-sur-Yvette, France; 2 Department of Earth System Science, Fukuoka University, Fukuoka, 814-0180 Japan; 3 School of Biological Sciences, University of Bristol, 24 Tyndall Avenue, Bristol, BS8 1TQ UK

**Keywords:** Olfactory system, Entomology, Chemical ecology

## Abstract

Olfaction is a crucial sensory modality underlying foraging, social and mating behaviors in many insects. Since the olfactory system is at the interface between the animal and its environment, it receives strong evolutionary pressures that promote neuronal adaptations and phenotypic variations across species. Hornets are large eusocial predatory wasps with a highly developed olfactory system, critical for foraging and intra-specific communication. In their natural range, hornet species display contrasting ecologies and olfactory-based behaviors, which might match to adaptive shifts in their olfactory system. The first olfactory processing center of the insect brain, the antennal lobe, is made of morphological and functional units called glomeruli. Using fluorescent staining, confocal microscopy and 3D reconstructions, we compared antennal lobe structure, glomerular numbers and volumes in four hornet species (*Vespa crabro*, *Vespa velutina*, *Vespa mandarinia* and *Vespa orientalis*) with marked differences in nesting site preferences and predatory behaviors. Despite a conserved organization of their antennal lobe compartments, glomeruli numbers varied strongly between species, including in a subsystem thought to process intraspecific cuticular signals. Moreover, specific adaptations involving enlarged glomeruli appeared in two species, *V. crabro* and *V. mandarinia*, but not in the others. We discuss the possible function of these adaptations based on species-specific behavioral differences.

## Introduction

The genus *Vespa* (Hymenoptera, Vespidae) comprises 22 species of large eusocial wasps commonly called hornets^1^. These insects are mainly distributed from temperate to tropical regions of South East Asia, sometimes in high density, so that high intra-and inter-specific competition occurs in many regions^2–4^. Hornet colonies grow and decline according to an annual life cycle. Usually, a single mated queen founds a paper nest to lay the eggs that will produce the first workers^2^. As in other social Hymenoptera, all individuals of the colony cooperate by performing different tasks, thanks to a complex communication system involving pheromones^5–9^. Hornets are generalist predators which prey on numerous insect species to supply their larvae with proteins. Consequently, they play a key role in the regulation of insect populations^10–13^. Lastly, they are known to be efficient invasive species, as shown by several recent invasion events^14–16^.

Strong interspecific competition can be severe, inducing significant evolutionary pressures on insects’ biology and behaviors. In tropical regions it has been observed that cohabiting species display an asynchronous development of their colonies which reduces competition for resources^2^. In addition, hornets display different preferences for nesting sites. Species like *Vespa velutina* build their nests preferentially in the trees, under treetop branches^14,17^, whereas *Vespa crabro* build theirs lower, in tree hollow cavities^2,18^ and *Vespa mandarinia* as well as *Vespa orientalis* nest underground. Hornets have also adopted remarkable strategies to overcome competition for nest foundation. Using chemical camouflage, *V. dybowskii* queens are able to perform social parasitism by usurping the nests of *V. crabro* and *V. simillima*^19,20^. In addition some species have colonized new environments, in which feeding resources and the competition to obtain them differ, exerting in turn distinct evolutionary pressures on predatory behaviors. Contrasting hunting strategies, such as specialization on a particular prey type or the recruitment of nestmates to food sources have been reported^21^. For instance, *V. tropica* hunt almost exclusively Polistes wasps, and conduct massive raids on their nests^21^.

Cooperative hunting is however more or less pronounced depending on the hornet species, ranging from *en masse* predation (e.g. *V. mandarinia*), in which scouts actively recruit nestmates with pheromones to attack insect colonies^22,23^, to solitary hunters locating food sources independently of other nestmates (e.g. *V. crabro*^24^), with intermediate cases of group predation (e.g. *V. velutina*^25^, *V. orientalis*^26^).

All these examples show that phylogenetically close species, such as different *Vespa* species, can acquire different behavioural traits to deal with specific ecological challenges. Because olfaction is pivotal for intraspecific communication, mating and foraging behaviors in hornets^9^, adaptations of their olfactory systems could underlie these ecological and behavioural variations.

In insects, odorant molecules are detected by olfactory sensory neurons (OSN) on the antennae, which project to a primary olfactory structure, the antennal lobe^27^ (AL). This structure is made of morphological and functional units called glomeruli, which individually gather inputs from a given type of OSN^28–30^. Recently, an increased effort has been made to describe and compare antennal lobe organization across species in Diptera^31–34^, Coleoptera^35–38^, Lepidoptera^39–44^ and Hymenoptera^45–51^. The AL of social Hymenoptera stands out as particularly complex as it contains a high number of glomeruli, compartmentalized in clusters formed by distinct bundles of OSN axons^52–56^. A differential investment in terms of numbers of glomeruli and glomerular clusters has already been observed across different Hymenoptera, possibly reflecting diverse demands on olfactory performance in this insect order^45,47–49,56–58^. Moreover, in males, but also in workers of some hymenopteran species, strongly enlarged glomeruli called ‘macroglomeruli’ are involved in the detection and processing of pheromonal signals^46,47,59–61^. An increased glomerular volume is thought to correlate with an expanded number of sensory neurons targeting the corresponding glomerulus^62–65^. Thus, the presence of macroglomeruli suggests the existence of neuronal adaptations for the enhanced detection of particularly relevant odorants in a given species.

So far, in Hymenoptera, previous comparative studies mostly concentrated on ants (Formicidae). In the present work, we addressed this question in the Vespidae, by comparing the antennal lobe organization of four hornet species of the genus *Vespa*. These species differ in ecology, nesting site preference and predatory behavior, which may have given rise to diverse neuronal adaptations in their olfactory system. We first assessed the variability in AL compartments across species and evaluated the number of glomeruli. Then, we measured and compared across species the volumes of glomeruli, asking if macroglomeruli exist in the AL of workers.

## Results

### Antennal lobe organization

**Figure 1:**
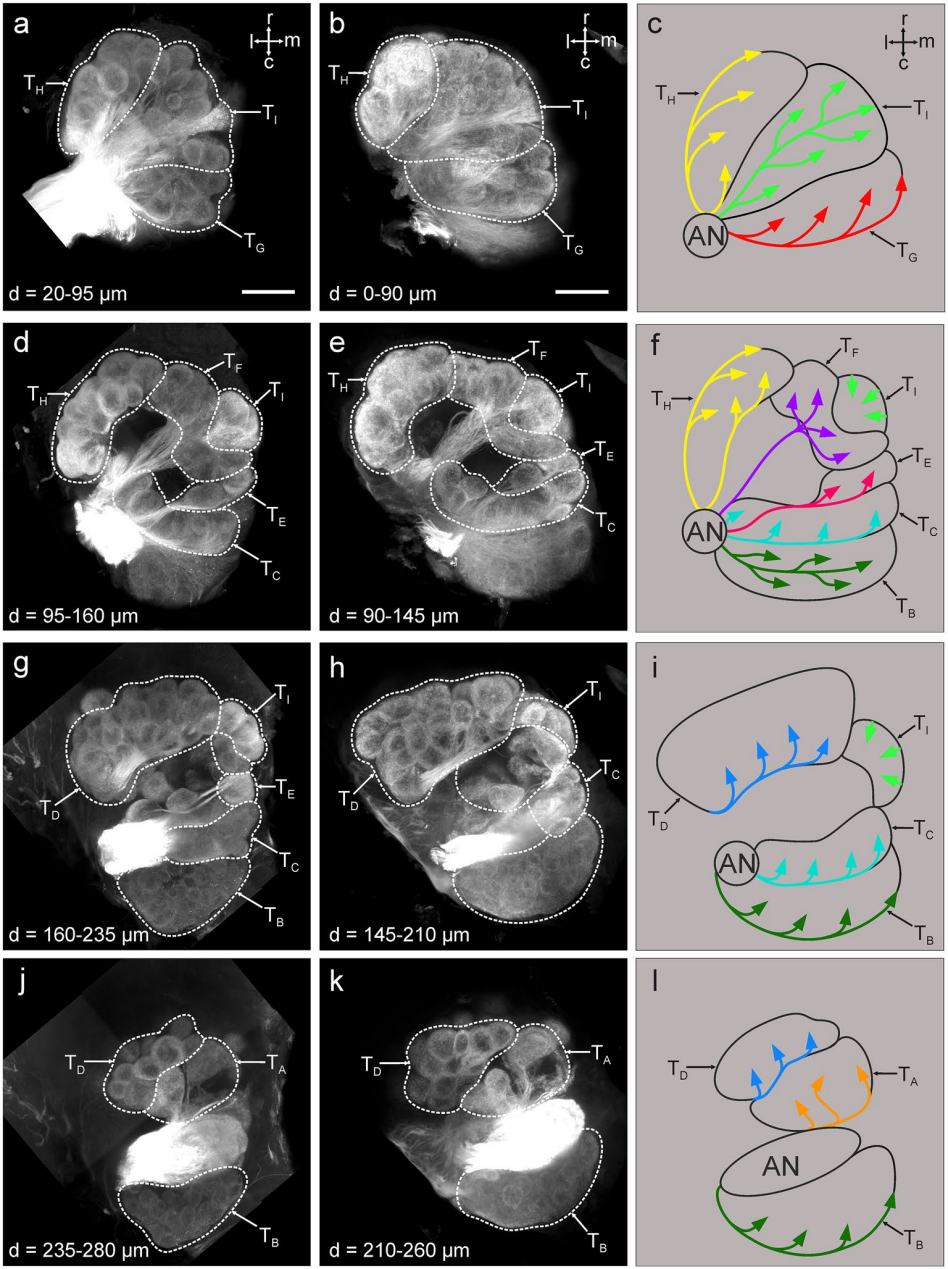
Antennal lobe organization in hornets. Projection view starting from the ventral surface to the dorsal surface of a right antennal lobe after mass staining of the antennal nerve in *V. velutina* (**a**, **d**, **g**, **j**) and *V. crabro* (**b**, **e**, **h**, **k**). The antennal lobes are similarly organized as both species exhibit 9 olfactory sensory tracts (T_A_–T_I_; **c**, **f**, **i**, **l**) which innervate similar clusters of glomeruli in both species. r, rostral; c, caudal; m, medial; l, lateral. All directions follow the neuraxis. Depth (d) from the ventral surface is indicated on each panel. The scale bars in (**a**) (applies to **d**, **g**, **j**) and (**b**) (applies to **e**, **h**, **k**) correspond to 100 µm.

In previous work, we extensively described the olfactory pathway of the hornet *V. velutina* and showed that the antennal lobe is compartmentalized in a number of conspicuous glomerular clusters subtended by OSN axon bundles^56^ (Fig. 1). To assess the similarity of this organization in other *Vespa* species, we injected a fluorescent tracer into the antennal nerve of *V. crabro* workers and observed the antennal lobe using confocal microscopy*.* This procedure revealed a very similar organization of the AL in *V. crabro* (n = 4) and in *V. velutina* (n = 6). In both species, the antennal nerve separates at the entrance of the AL into 9 OSN bundles (Fig. 1). These bundles follow different trajectories in, or around, the AL of the two species. They give rise to 9 clusters of glomeruli, which were termed T_A_-T_I_ from the most dorsal to the most ventral (a detailed description of each cluster is available in^56^). These 9 clusters were found at similar locations in the ALs of *V. crabro* and *V. velutina*, and contained glomeruli with similar shapes and sizes.

**Figure 2:**
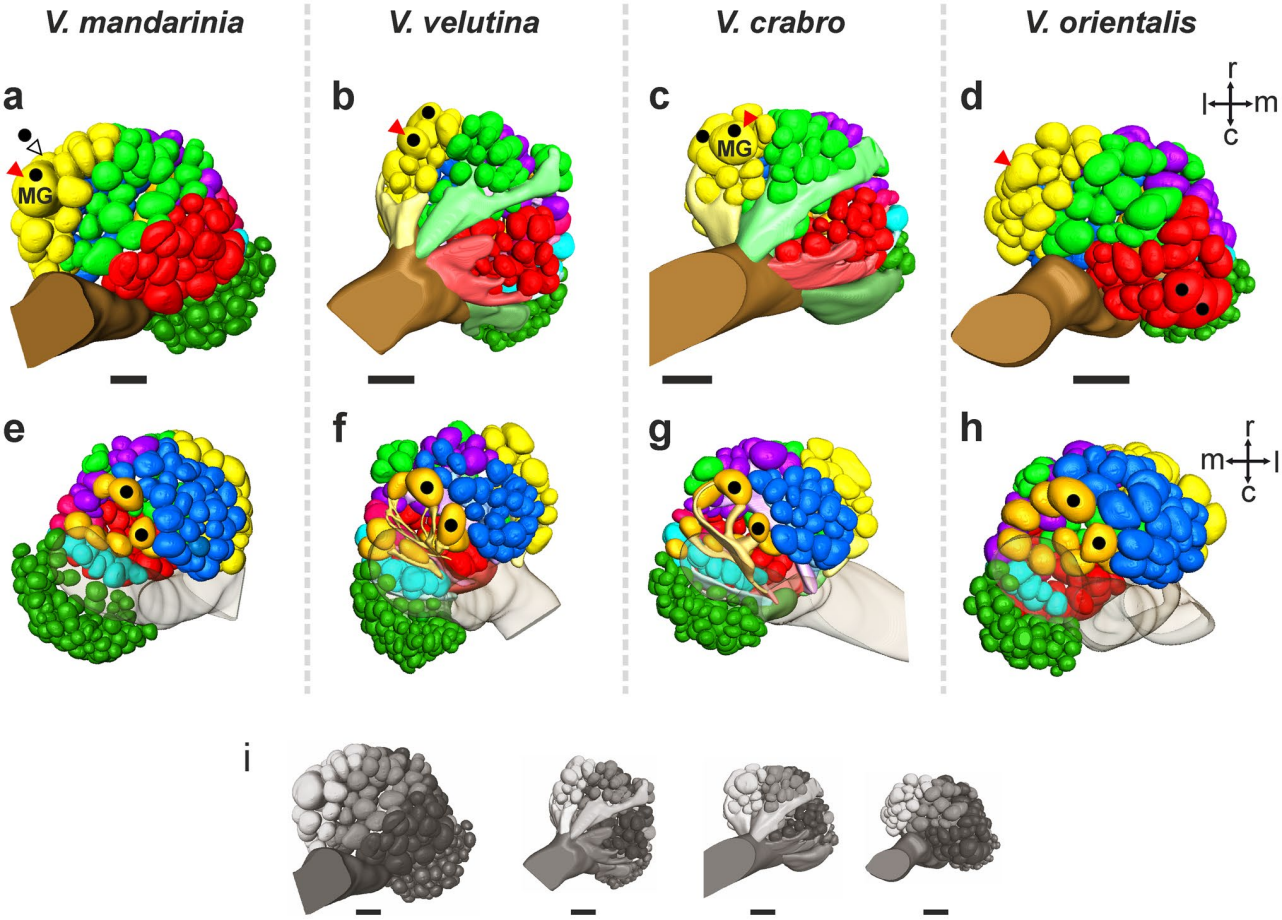
3D reconstructions of the antennal lobes of four hornet species. 3D models of the antennal lobes of *V. mandarinia* (**a**, **e**), *V. velutina* (**b**, **f**), *V. crabro* (**c**, **g**) and *V. orientalis* (**d**, **h**) are represented in a ventral view (**a**, **b**, **c**, **d**) or in a dorsal view (**e**, **f**, **g**, **h**). All lobes are scaled to the same size in (**a**–**h**) for easier comparison (scale bar = 100 µm). The glomerular clusters are colour-coded according to their homology across species, using the same colors as the subtracts in Fig. 1 (orange: T_A_; dark green: T_B_; light blue: T_C_; dark blue: T_D_; Pink: T_E_; purple: T_F_; red: T_G_; yellow: T_H_; light green: T_I_). Black dots indicate the four largest glomeruli in the antennal lobe of each species. The red triangle shows a possibly homologous glomerulus in the 4 species. MG stands for macroglomerulus and relates to the volumetric measures shown in Fig. 4. For reference, all lobes are represented according to their true relative sizes in (**i**) (scale bar = 100 µm).

Similarly, our stainings of *V. orientalis* and *V. mandarinia* ALs fit with these observations, leading us to use a common nomenclature for presumably homologous sensory tracts in all these species (Figs. 1 and 2, T_A_–T_I_^56^). Although global staining with bath-applied Lucifer yellow does not reveal OSN bundles as clearly as with tracer injection, we could compartmentalize the ALs of *V. orientalis* (n = 3) and *V. mandarinia* (n = 3) by additionally using recognizable glomerular features such as their relative size, shape and location. For instance, the large glomeruli in the most dorsal region of the AL (T_A_) were easily observed in all species (orange glomeruli in Fig. 2e–h). Also, the T_B_ cluster was easily identifiable as a dorsal cluster, partly segregated from the main AL (dark green in Fig. 2e–h). We conclude from these observations that the ALs of these four hornet species share the same organization.

### Number of glomeruli

**Figure 3:**
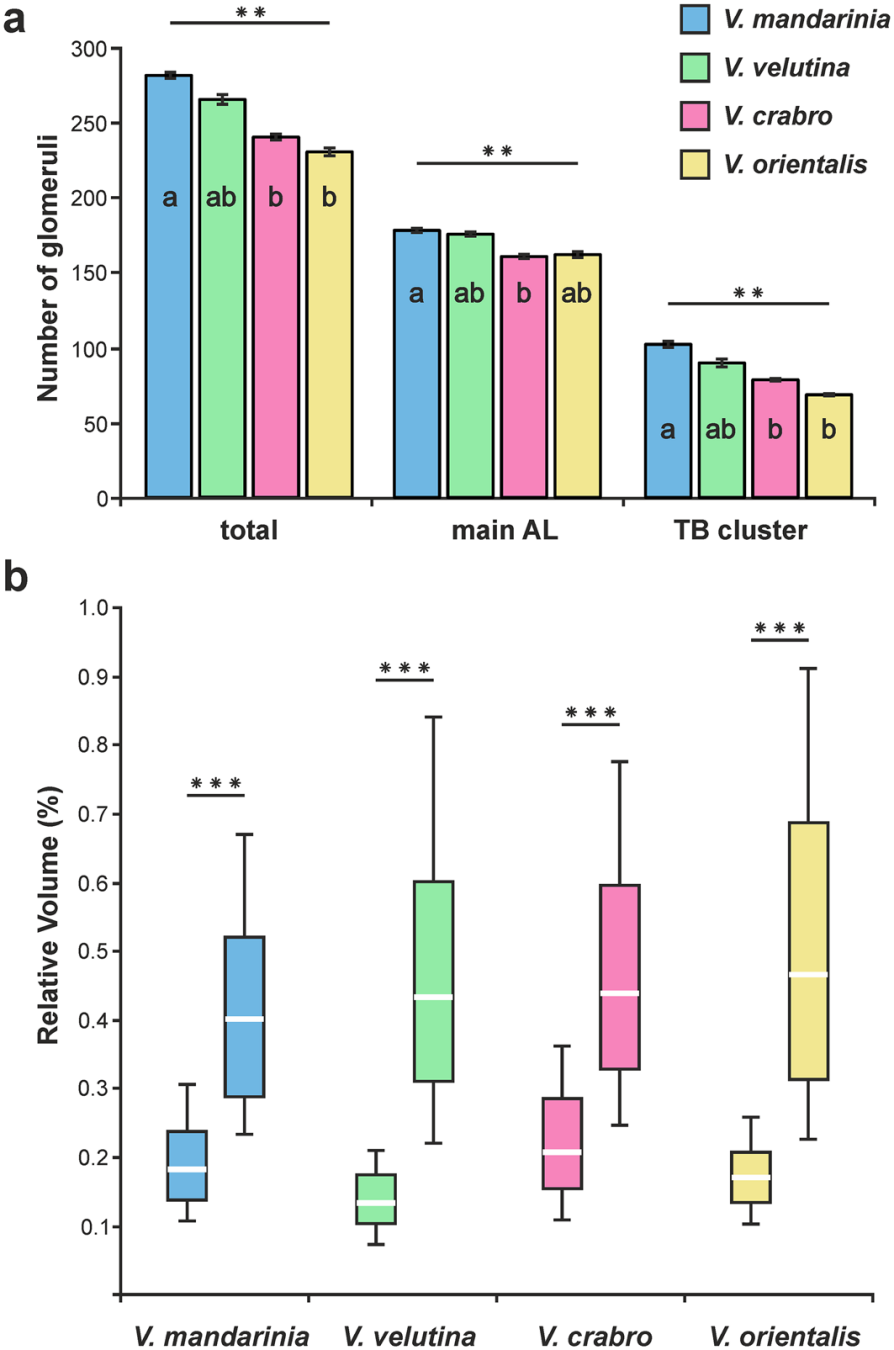
Number and volume of olfactory glomeruli in four hornet species. (**a**) Number of glomeruli in the whole antennal lobe (total) or its two main compartments (main AL and T_B_ cluster) in *V. mandarinia* (blue), *V. velutina* (green), *V. crabro* (pink) and *V. orientalis* (yellow). Significant differences are found within both compartments (Kruskal–Wallis test, **p < 0.01). Different letters indicate significant differences in post hoc Dunn tests (*p* < 0.05). (**b**) Box plots showing the relative volume of the T_B_ glomeruli (left box) compared to glomeruli of the main AL (right box) for each of the four hornet species. In all cases, T_B_ glomeruli are smaller than other glomeruli.

We assessed the number of glomeruli found in the ALs of the four hornet species. It ranged from 231 ± 3.6 (mean ± SD, n = 3) glomeruli in *V. orientalis* to 281 ± 2.9 (n = 3) glomeruli in *V. mandarinia* with intermediate cases at 265 ± 7.2 glomeruli in *V. velutina* (n = 7) and 240 ± 2.9 glomeruli in *V. crabro* (n = 4) (Fig. 3a and Table 1). The number of glomeruli was heterogeneous among the 4 hornet species (Kruskal–Wallis test, H = 14.6, *p* < 0.01). More precisely, the number of glomeruli in *V. crabro* and *V. orientalis* was significantly lower than that in the giant hornet *V. mandarinia* (Dunn test, Z = 2.722, *p* < 0.05 and Z = 3.395, *p* < 0.01, respectively).

**Table 1:** AL volume and glomeruli numbers in four hornet species.

Species	Whole AL		Main AL		T_B_ cluster	
	Volume	Nb glom	Volume	Nb glom	Volume	Nb glom
*V. mandarinia*	21.9 ± 3.9	281 ± 2.9	17.5 ± 3.2 (80.0%)	178 ± 2.3 (63.4%)	4.4 ± 0.7 (20.0%)	103 ± 2.6 (36.6%)
*V. velutina*	10.4 ± 3.0	265 ± 7.2	9.1 ± 2.6 (87.3%)	176 ± 3 (66.3%)	1.3 ± 0.4 (12.7%)	90 ± 6.9 (33.7%)
*V. crabro*	8.2 ± 1.2	240 ± 2.9	6.7 ± 1.1 (81.7%)	162 ± 2.7 (67.1%)	1.5 ± 0.2 (18.3%)	79 ± 1 (32.9%)
*V. orientalis*	12.2 ± 0.8	231 ± 3.6	10.7 ± 0.6 (87.7%)	162 ± 3.0 (70.4%)	1.5 ± 0.2 (12.3%)	68 ± 0.6 (29.6%)

The table shows AL volumes (calculated as the sum of the volumes of all glomeruli; × 10^6^ µm^3^) and the numbers of glomeruli (Nb, ± standard deviation) in all studied species. Values are also given separately for two AL compartments (T_B_ cluster and the main AL), along with the respective proportion of the whole AL, shown within brackets.

We next focused on one particular subsystem of the hornet AL. As mentioned above, the T_B_ forms a conspicuous cluster, which is partly segregated from the main part of the AL and is assumed to process cuticular hydrocarbon information for intra- and interspecific discrimination (hornets^66^; ants^67–72^). We thus analyzed separately the number of glomeruli contained in the T_B_ cluster and in the main AL for each species (Table 1). We found that both AL compartments contained significantly different numbers of glomeruli across species (Main AL: Kruskal–Wallis test, H = 12.2, *p* < 0.01; T_B_: H = 14.2, *p* < 0.01). On average, the main AL contains about 162 glomeruli in *V. crabro* (± 2.7) and *V. orientalis* (± 3.0) whereas about 176 ± 3 glomeruli where counted in *V. velutina* and 178 ± 2.3 in *V. mandarinia*. A significant difference appeared between *V. crabro* and *V. mandarinia* (Dunn test, Z = 2.733, *p* < 0.05). The T_B_ cluster contains about 68 ± 0.6 glomeruli in *V. orientalis*, 79 ± 1 glomeruli in *V. crabro*, 90 ± 6.9 glomeruli in *V. velutina* and 103 ± 2.6 glomeruli in *V. mandarinia*, with a significant difference when comparing *V. mandarinia* with either *V. crabro* or *V. orientalis* (Dunn test, Z = 2.658, *p* < 0.05 and Z = 3.395, *p* < 0.01, respectively). Thus, both in the T_B_ subsystem and in the main AL, different hornet species present different numbers of glomeruli.

To assess hornets’ investment in the T_B_ subsystem in terms of number of functional units (glomeruli), we compared their number in this system relative to the whole AL. The relative investment in the T_B_ cluster varied significantly across species (Kruskal–Wallis test, H = 11.9, *p* < 0.01). It represented from 29.6% of the total number of glomeruli in *V. orientalis* to 36.5% in *V. mandarinia*, with a significant difference between these two species (Dunn test, Z = 3.395, *p* < 0.01). We conclude that the relative investment in the T_B_ cluster differs across *Vespa* species.

### Volume of glomeruli

We used 3D models to measure the volume of each glomerulus in the ALs of the four *Vespa* species. In hornets, the size of individuals varies greatly between species, affecting the absolute volume of neural structures. Here, the AL of the giant hornet *V. mandarinia* was on average twice as large (21.9 × 10^6^ µm^3^) as those of the three other species (ranging from 82.9 × 10^5^ to 12.2 × 10^6^ µm^3^; Fig. 2i). To overcome such size differences, we calculated the relative volume of each glomerulus according to the sum of all AL glomeruli, as a measure of hornets’ investment in this particular morphological unit. This first allowed us to compare across species the relative volume of the T_B_ subsystem. In all species, the relative volume of T_B_ glomeruli was smaller (on average between 0.14 and 0.23%, Fig. 3b) than the relative volume of glomeruli from the main AL (on average between 0.45 and 0.54%; Wilcoxon test, Z = 3.621, *p* < 0.001). The whole T_B_ system represented ~ 20% of the whole AL volume in *V. mandarinia*, 18% in *V. crabro*, but only about 12.5% in *V. velutina* and *V. orientalis* (Table 1). These proportions were statistically heterogeneous among species (Kruskal–Wallis test, H = 12.41, *p* < 0.01), with a significant difference between the two extreme cases, *V. orientalis* and *V. mandarinia* (Dunn test, Z = 2.748, *p* < 0.05).

**Figure 4:**
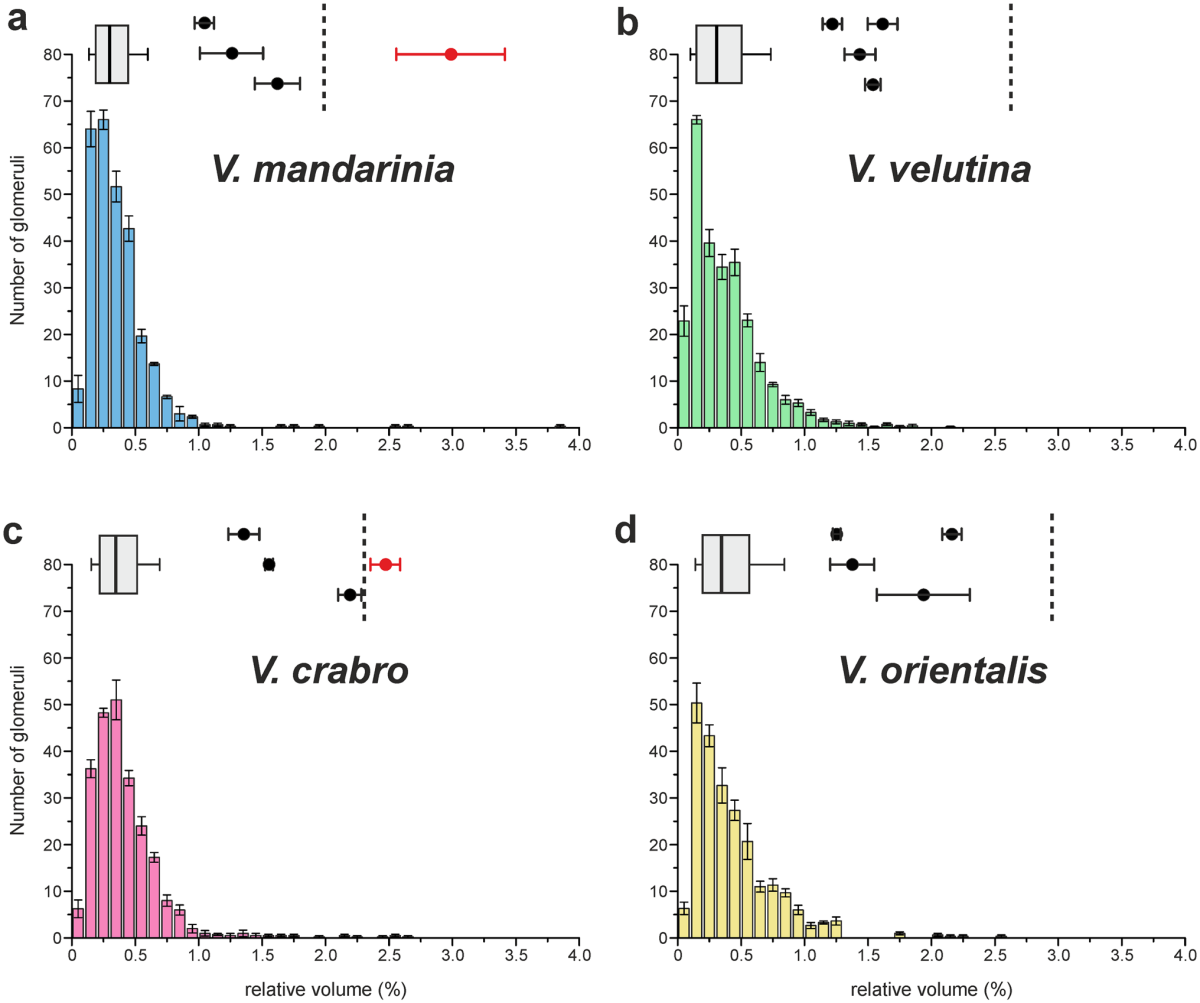
Distribution of glomerular volumes in four hornet species. Distribution of all glomerular volumes in the reconstructed antennal lobes of *V. mandarina* (**a**, n = 3), *V. velutina* (**b**, n = 7), *V. crabro* (**c**, n = 4) and *V. orientalis* (**d**, n = 3). The box plots above each distribution show the median and interquartile range (25–75%) of glomerular volumes whereas whiskers show the 10th and 90th percentiles. The average volumes of the four largest glomeruli of each species are shown with black circles and the statistical macroglomerulus threshold is represented by a dotted line. Only the antennal lobes of *V. mandarinia* (**a**) and *V. crabro* (**c**) contain a glomerulus that exceeds this threshold. This macroglomerulus is represented with a red circle (labeled MG in Fig. 2).

The calculation of the relative glomerular volumes also allowed us to assess the possible presence of macroglomeruli in the hornet AL. In the four species, most of the glomeruli displayed a relatively similar size, each representing less than 1% of the total AL (Fig. 4). Nonetheless, within each lobe, a few conspicuously large glomeruli were found. We focused on the four largest glomeruli found in the AL of each species (black dots in Fig. 2a–h). In all species, two of these were located in the dorsal cluster T_A_ (black dots in Fig. 2e–h). In three of the species, *V. mandarinia*, *V. velutina* and *V. crabro*, the next two large glomeruli were found on the ventral side, in the T_H_ cluster (black dots in Fig. 2a–c). All these glomeruli were found at similar locations across different species and might be homologous (synapomorphic). *Vespa orientalis* differed from the other species because two of the four largest glomeruli were rather found in the T_G_ cluster, on the ventral side (black dots in Fig. 2d). We then asked whether any of these four largest glomeruli per species could be considered as macroglomeruli, based on a volumetric threshold that defines outliers according to the distribution of glomerular volumes (Fig. 4). We found that only the largest glomerulus located on the ventral AL surface of *V. mandarinia* and *V. crabro* passes the threshold and can be classified as a macroglomerulus (‘MG’ in Fig. 2a, c and outliers in Fig. 4a, c). We conclude that only the ALs of *V. mandarinia* and *V. crabro* workers contain a macroglomerulus.

## Discussion

The goal of this study was to investigate possible neuronal adaptations within the antennal lobes of different hornet species in relation with differences in their biology and behavior. We found that the general organization of the AL, such as its compartmentation in nine recognizable clusters of glomeruli, is conserved across species. However, two phenotypical traits were found to be variable. First, the number of glomeruli varied across species, especially within a particular cluster of glomeruli, the T_B_, thought to be involved in nestmate discrimination. Second, although the relative distribution of glomerular volumes was relatively stable across species, a macroglomerulus (MG) was found in two of the investigated species, *V. crabro* and *V. mandarinia*.

### Species-specific differences in AL composition

The primary olfactory processing center, the AL, is made of dense synaptic neuropil units which individually gather all the OSNs expressing a given olfactory receptor (OR)^28,30,73^. Consequently, the number of glomeruli closely correlates with the number of olfactory receptor genes expressed within a species and is thought to reflect the general olfactory discrimination power. For instance, consistently with the one-receptor/one-glomerulus, ~ 170 functional OR genes were annotated in the honeybee, which has about 165 olfactory glomeruli in the antennal lobe^74,75^. Social insects deal with a highly complex olfactory environment in which they must be able to discriminate among numerous different odorants. Interestingly, important expansions of chemoreceptor gene repertoires have been observed in Hymenoptera, correlating with an expansion of the number of AL glomeruli found throughout this insect order^76^. We found that the olfactory system of hornets, with at least 230 glomeruli in all investigated species, fits with the general trend observed in Hymenoptera. Although hornet genomes are not available yet, we expect that a similarly high number of functional OR genes will be found.

We also observed high differences in glomeruli numbers across species (50 glomeruli difference between *V. mandarinia* and *V. orientalis*). Important differences in glomeruli numbers within the same genus were also observed in other social Hymenoptera, the leaf cutting ants (*Atta* sp. 116 glomeruli ; *Acromyrmex* sp. 108 glomeruli^47^). This contrasts with the situation in some other insect orders, in which very little variance in glomeruli numbers was found. For instance, in Lepidoptera, only minor variations in glomerular numbers were described, even between distant families^41^.

The hymenopteran AL presents a remarkable organization with multiple compartments, possibly homologous in different clades^54,56,77^. The most conspicuous subdivision of the hornet AL is the T_B_ cluster, which exhibits remarkable resemblance in ants and vespid wasps^66^. This cluster, which is partly segregated from the main AL, is thought to process cuticular hydrocarbon information detected at the level of basiconic sensilla (ants^67–69^; hornets^66^). Accordingly, basiconic sensilla shelter sensory neurons which express members of a particular subfamily of OR genes with a 9-exon structure^70–72^. One could speculate that variable demands on inter- and intraspecific discrimination between hornet species could promote adaptive shifts within the T_B_ cluster. We therefore separately assessed the numbers of glomeruli in the T_B_ cluster and in the main AL. We found that although the number of glomeruli in the main AL slightly (but significantly) varied across species, larger differences among species were found for the T_B_ cluster, with a higher investment in *V. mandarinia* than in *V. orientalis* or *V. crabro*. If the T_B_ cluster houses OSNs carrying 9-exon ORs, as proposed in ants, this results possibly suggests important variations in the number of these genes in the genomes of hornet species. Consistently with this idea, a high evolutionary rate for 9-exon OR genes has been observed in a group of closely related social species, the Polistes paper wasps^78^. This could thus be a general trend of social wasps of the *Vespinae and Polistinae* families.

The T_B_ cluster showed a high diversification, with *V. mandarinia* having 51% more glomeruli in their T_B_ subsystem (103 glomeruli) than *V. orientalis* (68 glomeruli). Interestingly, hornet species which have their natural range in Asia (*V. mandarinia and V. velutina*), which implies a high density of hornet species^4,21^, also have more T_B_ glomeruli than the Europe dwelling species *V. crabro* and *V. orientalis.* These species may experience lower interspecific (possibly also intraspecific) competition in western countries putting less demand on olfactory discrimination. A separate subspecies of *V. crabro* (*V. crabro flavofasciata*) which forms large colonies is found in Japan, with accordingly high species density^79^. It would be especially interesting to compare the numbers of T_B_ glomeruli in both *V. crabro* subspecies to investigate a potential adaptation to high intra- and interspecific competition.

### Worker macroglomeruli

Many studies have shown sex and caste polymorphism in glomerular volume within the same species (bees^50,59,80,81^; ants^46–48,82,83^). The size of a glomerulus generally correlates with the number of OSNs terminating in this glomerulus, and a large number of OSNs is thought to enhance the detection sensitivity of its associated compounds^62,64,65^. Thus, glomerular volume is a well-established measure of the system’s sensitivity and the presence of hypertrophied glomeruli generally indicates the importance of particular compounds in the biology of a given species. The best known illustrations of hypertrophied glomeruli are the macroglomeruli found in male antennal lobe of numerous insects such as moths, butterflies, cockroaches, honeybees, hornets and ants^51,56,59,63,82,84,85^. In these species, the male-specific macroglomeruli are related to the detection and processing of sex pheromones. Within Hymenoptera, macroglomeruli have also been found in females but their function is often unclear^46,80,81^. A well described example is the macroglomerulus found in the large workers of some leaf cutting ants (*Atta* sp. and *Acromyrmex* sp.), which is involved in the processing of a trail pheromone component^47,61^. In the present study, we found a ventral glomerulus with widely differing volumes in the four hornet species (red triangles in Fig. 2a–d). Due to its enlarged size in two species (*V. mandarinia* and *V. crabro*) and following the use of a standard statistical threshold, it classified as a worker macroglomerulus. This suggests a progressive size increase in some of these species, and a putative adaptation for the detection of particularly relevant compounds in their biology. It should be pointed out that individual experience may affect glomerular volume, possibly affecting our conclusions. For instance, in honey bees, appetitive experience at different ages produced both increases and decreases in the volume of particular antennal lobe glomeruli^86,87^, while in *Drosophila*, simple odour exposure was found to diminish glomerular volume^88^. In the present case, we could not control the previous experience of our wild caught hornets, and we cannot exclude that such effects could affect the classification of the largest glomeruli above or below the macroglomerulus threshold. However, given the relatively low level of volumetric change induced by experience in previous studies (maximum 20%), most of our classifications should remain unaffected. As shown in Fig. 4, the presence of a macroglomerulus in *V. mandarinia*, as well as the lack thereof in *V. velutina* and *V. orientalis* appear beyond such possible effects. In contrast, because the volumes of the 2 largest glomeruli in *V. crabro* lie close to the threshold, the presence of 0, 1 or 2 macroglomeruli in this species are possible.

At this stage, without functional data, we can only speculate about the possible function of hornet workers’ putative macroglomeruli. *Vespa crabro* and *V. mandarinia* are both typical cavity-nesting species which could use trail pheromone for advertising the nest entrance^2,18^. However, *V. orientalis,* which does not feature such a macroglomerulus, also nests within cavities^89^. Thus, unless interspecific differences in processing strategies exist (for instance, including a strategy based on a combinatorial code without the use of macroglomeruli, such as in the ant *Componotus floridanus*^54^), the nesting behavior of hornets cannot explain the presence of a macroglomerulus in some species and not in others.

The worker macroglomeruli could also be involved in the processing of alarm pheromone^23,90^. However, like for the trail pheromone, the use of alarm pheromone is not exclusive to *V. crabro* and *V. mandarinia*^8,91^. Nevertheless, worker macroglomeruli could enhance the sensitivity of these species to their alarm pheromone. An alternative hypothesis relates to pheromones involved in hornets’ recruitment behavior. *Vespa mandarinia* performs *en masse* predation and is able to recruit nestmates to food sources by means of recruitment pheromones^22,23^. However, *V. crabro,* which has a widely differing, mostly solitary, predation behavior also displays a macroglomerulus. Consequently, we are currently not able to provide a solid hypothesis based on present knowledge of these hornets’ behavior. Further functional data aiming to find the receptive range of OSNs targeting the hypertrophied glomeruli in the two species might help understand its function.

Finally, our results show that the volume of glomeruli is a highly variable phenotypic trait in hornets. This suggests that evolutionary pressures might act on the number of OSN targeting a given glomerulus, finely tuning the detection sensitivity of the hornets’ olfactory system to particular compounds.

## Materials and methods

### Animals

Four species belonging to the *Vespa* genus were chosen. Workers of two species were collected in France: European hornets, *V. crabro*, were trapped in Gif-sur-Yvette, and yellow-legged hornets *V. velutina* were caught in Villenave d’Ornon. Workers of the giant hornets, *V. mandarinia* were trapped in Fukuoka, Japan, and those of the oriental hornets, *V. orientalis*, were captured around Porto-Heli, Greece. All species were easily distinguishable based on their distinct sizes and body colorations.

### Staining procedure

To investigate the glomerular organization of the AL, antennal sensory neurons were stained anterogradely in the two species collected in France, *V. crabro* and *V. velutina*. The antennal nerve was exposed by removing a piece of cuticle at the scape. Then, the antennal nerves were severed with a glass electrode loaded with crystals of fluoro-ruby (Tetramethylrhodamine dextran, 10 000 MW, D-1817; Invitrogen, Eugene, OR; in 2% Bovine Serum Albumin). The preparation was covered with saline solution (130 mM NaCl, 6 mM KCl, 4 mM MgCl_2_, 5 mM CaCl_2_, 160 mM sucrose, 25 mM glucose, 10 mM HEPES, with PH = 6.7) and kept in a dark room to let the dye diffuse. The next day, brains were dissected out and immediately fixed in 4% PFA solution for 24 h.

After the capture of *V. mandarinia* and *V. orientalis*, the head of each insect was immediately separated from the body and immersed in 500 μL of fixative solution (4% paraformaldehyde in PBS) containing 1 µL of 4% Lucifer yellow (Lucifer Yellow CH, Potassium Salt, L-1177; Invitrogen, Eugene, OR). Samples were kept in this solution for 5 to 7 days before dissection of the brains.

Thus, staining varied among species but the fixation and dehydration steps were the same (see below). To assess a potential bias of the different staining protocols, we performed both approaches in *V. velutina* workers. Both techniques provided good contrast and produced consistent 3D models and glomerular distributions. In addition, we did not observe any systematic bias in glomerular numbers or relative sizes depending on staining procedure.

### Brain preparation and confocal microscopy

Brains were removed from the fixative solution and washed 3 times in 0.01 M of PBS (10 min each). Brains were then dehydrated in ascending concentrations of ethanol (50, 70, 90, 95 and 3 × 100% for 10 min each) and clarified in methylsalicylate (Sigma-Aldrich, Steinheim, Germany) for at least 3 days. The samples were then mounted on aluminum slides with a central hole filled with methylsalicylate and covered by thin coverslips on both sides.

Antennal lobes were scanned with a laser-scanning confocal microscope (LSM-700; Carl Zeiss, Jena, Germany). Using a water immersion objective (20 × plan-apochromat 1.0 NA), optical sections were acquired at 1 µm intervals (z), with a resolution ranging from 0.52 to 0.69 µm/pixels (x,y), depending on the size of the AL of each species. Given the large size of the hornet AL, complete scans were obtained by stitching adjacent “tiles” (512 × 512 pixels) of optical sections with the tile function of the ZEN software (Carl Zeiss, Jena, Germany). Fluoro-ruby labeled neurons were visualized using a 555 nm solid-state laser, while Lucifer yellow was excited at 488 nm.

### Image processing and 3D reconstruction

Confocal image stacks were saved as LSM files and processed using ImageJ software and the bio-formats plugin (LOCI). Contrast and brightness were adjusted for each series of optical sections, which were then imported into a three-dimensional analysis software (AMIRA 5.4.3, VSG, Berlin, Germany). Glomeruli were reconstructed by manual labeling in three orthogonal plans (xy, xz and yz) and the 3D model of each glomerulus was obtained using the *wrap* function of AMIRA. When the antennal nerve was stained, it was possible to assign each glomerulus to a glomerular cluster by closely following OSN bundles. In contrast, with Lucifer yellow staining, OSN bundles were less contrasted and antennal lobe compartmentalization also used glomerular position, shape and relative size. However, irrespective of the staining method, the cluster termed T_B_ could be unambiguously delineated thanks to its typical features (slightly detached from main AL, with smaller, tightly packed glomeruli). All directions in this study are given according to the neuraxis.

### Volume measurement and statistical analysis

The volume of glomeruli was measured on the basis of their 3D model using AMIRA software. To overcome the problem of antennal lobe size variability in different individuals or across species, the volume of each glomerulus was normalized with respect to the size of the AL (calculated as the sum of all glomerular volumes; relative volume in Figs. 3b and 4). For interspecific comparisons of glomerular numbers and volumes, non-parametric Kruskal Wallis tests were used. When significant, they were followed by a Dunn pairwise test, which includes a correction for multiple comparisons. These tests were performed with Statistica 10.

In order to determine the presence of macroglomeruli, the four largest glomeruli of each species were identified in all individuals and the mean relative volume was calculated for each homologous glomerulus. Then, a quantitative threshold that defines outliers according to 80% of the distribution of glomerular volumes was used: V_outlier_ > V_U_ + k (V_U_ – V_L_), where V_U_ is the upper percentile (90%) and V_L_ is the lower percentile (10%) of glomerular volume distribution. We used k = 3 as a conservative value that successfully categorized macroglomeruli in previous studies^50,51,56,61,81^. Thus, glomeruli whose volume was above this threshold were considered as macroglomeruli.
